# Teratogen-induced alterations in microRNA-34, microRNA-125b and microRNA-155 expression: correlation with embryonic p53 genotype and limb phenotype

**DOI:** 10.1186/1471-213X-10-20

**Published:** 2010-02-21

**Authors:** Keren Gueta, Natali Molotski, Natalie Gerchikov, Eyal Mor, Shoshana Savion, Amos Fein, Vladimir Toder, Noam Shomron, Arkady Torchinsky

**Affiliations:** 1 Department of Cell and Developmental Biology, Sackler School of Medicine, Tel Aviv University, Ramat Aviv, Tel Aviv, 69978, Israel; 2 Department of Biological Chemistry, the Weizmann Institute of Science, Rehovot, Israel

## Abstract

**Background:**

In a large number of studies, members of the microRNA (miRNA)-34 family such as miRNA-34a, miRNA-34b, miRNA-34c, as well as miRNA-125b and miRNA-155, have been shown to be regulators of apoptosis. The ability of these miRNAs to perform this function is mainly attributed to their ability to interact with the p53 tumor suppressor, which is a powerful regulator of the teratologic susceptibility of embryos. We chose to explore whether miRNA-34a/b/c, miRNA-125b and miRNA-155 may play a role in teratogenesis by using p53^+/- ^pregnant mice treated with cyclophosphamide (CP) as a model. We evaluated how CP-induced alterations in the expression of these miRNAs in the embryonic limbs correlate with embryonic p53 genotype and CP-induced limb phenotypes.

**Results:**

The limbs of p53 positive embryos were more sensitive to CP-induced teratogenic insult than the limbs of p53 negative embryos. The hindlimbs were more severely affected than the forelimbs. Robust miRNA-34a expression was observed in the fore- and hindlimbs of p53^+/+ ^embryos exposed to 12.5 mg/kg CP. The dose of 20 mg/kg CP induced almost a two-fold increase in the level of miRNA-34a expression as compared to that exhibited by p53^+/+ ^embryos exposed to a lower dose. Increased miRNA-34b and miRNA-34c expression was also observed. Of note, this dose activated miRNA-34a and miRNA-34c in the forelimbs of p53^-/- ^embryos. When embryos were exposed to 40 mg/kg CP, the expression pattern of the miRNA-34a/b/c was identical to that registered in the limbs of embryos exposed to 20 mg/kg CP. However, this dose suppressed miRNA-125b and miRNA-155 expression in the fore- and hindlimbs of p53^+/+ ^embryos.

**Conclusion:**

This study demonstrates that teratogen-induced limb dysmorphogenesis may be associated with alterations in miRNA-34, miRNA-125b and miRNA-155 expression. It also suggests for the first time that p53-independent mechanisms exist contributing to teratogen-induced activation of miRNA-34a and miRNA-34c. At the same time, teratogen-induced suppression of miRNA-125b and miRNA-155 expression may be p53 dependent. The analysis of correlations between the expression pattern of the tested miRNAs and CP induced limb phenotypes implies that miRNAs regulating apoptosis may differ from each other with respect to their functional role in teratogenesis: some miRNAs act to protect embryos, whereas other miRNAs boost a teratogen-induced process of maldevelopment to induce embryonic death.

## Background

Mature microRNAs (miRNAs) are non-coding RNAs composed of about 22-nucleotide, that attenuate gene activity posttranscriptionally by inhibiting effective mRNA translation of target genes. Silencing takes place through sequence-specific base pairing between the miR and its target mRNAs [1,2]. By now, hundreds of miRNAs have been detected [3] and some miRNAs have been shown to be essential for normal embryonic development, controlling developmental events such as neurogenesis, angiogenesis, and the formation of limbs, heart and muscles [4,5]. In parallel, studies in invertebrates and various types of cultured cells revealed the ability of some miRNAs to regulate cell proliferation and apoptosis [6,7]. These observations have formulated a basis to suggest that miRNAs may play an important role in cancer formation, acting both as oncogenes and tumor suppressors [8]. Remarkably, these observations also suggest that miRNAs may act as regulators of embryos' susceptibility to developmental toxicants (teratogens). Indeed, apoptosis and cell proliferations are critically important processes of normal embryogenesis [9]. Teratological studies have revealed that the appearance of teratogen-induced structural anomalies is often preceded by excessive apoptosis in embryonic structures that are destined to be malformed [10,11]. At the same time, teratogen-induced apoptosis is also often registered in embryonic structures that appear normal at birth [10,11]. This demonstrates that the embryo is able to compensate for teratogen-induced cell death and, hence, teratologic susceptibility of embryos depends not only on the mechanisms regulating apoptosis but also on mechanisms regulating cell proliferation.

Recently, a number of studies have provided compelling evidence that members of the miRNA-34 family (hereafter abbreviated as miRNA-34) such as miRNA-34a, miRNA-34b and miRNA-34c are direct transcription targets of the tumor suppressor protein p53, having the potential to regulate both apoptosis and cell proliferation [12]. The role of p53 as a regulator of teratological susceptibility of embryos has been demonstrated in studies with diverse teratogens such as benzo(a)pyrene [13,14], 2-chloro-2-deoxyadenosine [14], 4-hydroperoxycyclophosphamide [15], cyclophosphamide [16], ionizing radiation [17,18] and diabetes [19]. Quite a few genes have been demonstrated as mediators of p53- induced apoptosis and cell arrest [20,21], but those acting in teratogen-exposed embryos remain largely undefined. Therefore, our question was whether miRNA-34 may be among targets engaged by p53 to regulate teratologic susceptibility of embryos.

Two other miRNAs, miRNA-125b and miRNA-155 also seemed to be good candidates for the role of teratologic regulators. Specifically, our and others study with cyclophosphamide (CP) have revealed that excessive apoptosis is a major event in the pathogenesis of CP-induced process of maldevelopment [10,22]. p53 acts to intensify both CP-induced apoptosis and suppression of cell proliferation [16]. It also mediates CP -induced activation of caspase 3 and suppression of the transcription factor NF-kB DNA binding [16]. Furthermore, our recent work has implied that CP-induced suppression of NF-kB signaling may be linked to CP-induced apoptosis and suppression of cell proliferation [23]. In turn, miRNA-155 has been shown to regulate apoptosis and suggested to target caspases 3 and NF-kB signaling [24,25]. MiRNA-125b has been suggested to control the expression of the tumor necrosis factor alpha (TNFα) [26], a cytokine, acting as a powerful activator of NF-kB [27]. Notably, our studies have provided evidence suggesting that TNFα may determine sensitivity of mice to CP-induced teratogenic insult [28].

Given the potential involvement of miRNA-34, miRNA-125b and miRNA-155 in the mechanisms regulating teratologic susceptibility of embryos, we chose to explore whether CP alters the expression of the miRNAs in the embryonic limbs and how the alterations correlate with the embryonic p53 genotype and CP-induced limb phenotypes. We used CP-treated p53 heterozygous mice as a model and the fore- and hindlimbs of p53 knockout and p53 positive embryos as target embryonic structures.

## Results

### Reproductive performance of CP-treated mice

In females injected with the highest dose of CP (40 mg/kg), the level of postimplantation death of embryos exceeded 26% (Table 1). This was a significant increase when compared to that of females exposed to lower doses of CP and controls. In these females a trend to the departure from the Mendelian 25%:50%:25% genotype ratio (≈29%:60%:11%) due to the death of p53^-/- ^embryos was also registered. In mice exposed to lower doses of CP these indices did not differ significantly from those in controls (Table 1).

**Table 1:** Reproductive performance of p53^+/- ^females treated with cyclophosphamide (CP) and tested on day 16 of pregnancy.

	Groups of females			
**Indices**	**Control**	**CP (12.5 mg/kg)**	**CP (20 mg/kg)**	**CP (40 mg/kg)**

Number of pregnant females	8	10	14	10

Implantation sites/litter	66/8.3	84/8.4	106/7.6	83/8.3

Percent of resorptions (arcsine, mean ± SE)	6.1 (16.2 ± 3.4) ^a^	4.8 (15.4 ± 2.5) ^a^	13.2 (23.5 ± 3.6)^a^	26.5 (32.0 ± 2.0) ^b^

Number of live fetuses				

Total	62	80	92	61

p53^+/+^	18 (29%)	14 (17.5%)	16 (17.4%)	18 (29.5%)

p53^+/-^	27 (43.6%)	46 (57.5%)	48 (52.2%)	36 (59%)

p53^-/-^	17 (27.4%)	20 (25%)	28 (30.4%)	7 (11.5%)

Superscripts denote results of statistical analysis of values within a row. Values not sharing common superscripts are significantly different (the GT-2 test, p < 0.05).

### Teratogenic response to CP

As expected, the forelimbs and hindlimbs exhibited differential sensitivity to CP-induced teratogenic stimuli. Indeed, practically all fetuses of mice exposed to 40 mg/kg had digit anomalies of the fore- and hindlimbs (Table 2). At the same time, limb reduction anomalies of the hindlimbs but not of the forelimbs were registered in these fetuses. In females exposed to 20 mg/kg, the proportion of embryos having digit anomalies of the forelimbs was significantly lower than that of embryos exhibiting digit anomalies of the hindlimbs. Finally, ≈30% of embryos exposed to 12.5 mg/kg CP had digit anomalies of the hindlimbs but only single fetuses had malformed forelimbs (Table 2).

**Table 2:** Limb phenotypes exhibited by embryos of p53^+/- ^mice treated with CP

Doses of cyclophosphamide		12.5 mg/kg	CP 20 mg/kg	CP 40 mg/kg
Number of tested embryos		80	92	61

Type of anomalies		Fetuses with malformed limbs^§^		
	
	Forelimbs	6 (7.5%)	24 (26.1%)	59 (96.7%)
		17.7 ± 3.5^a^	32.4 ± 3.1^b^	73.0 ± 0.5 ^c^
Digit anomalies^1 ^arcsine, mean ± SE				
	Hindlimbs	23(28.8%)	66 (71.7%)	61 (100%)
		32.5 ± 3.5^a^*	55.9 ± 3.8^b^*	78.9 ± 0.3^c^

	Forelimbs	0	0	0
		9.9 ± 0.3	10.7 ± 0.4	11.0 ± 0.3
Limb reduction anomalies, arcsine, mean ± SE				
	Hindlimbs	0	24 (26.1%)	61 (100%)
		9.9 ± 0.3^a^	32.4 ± 3.1^b^*	78.9 ± 0.3^c^*

^1^Digit anomalies that were registered included syndactyly, ectrodactyly, adactyly. Limb reduction anomalies included amelia, apodia and hemimelia.

^§^No fetuses with digit and limb anomalies were found in litters of control mice. Superscripts denote results of statistical analysis of values within a row. Values not sharing common superscripts are significantly different (the GT-2 test, p < 0.05).

*Differences between the fore- and hindlimbs are statistically significant (Student's t-test, p < 0.05).

### Effects of p53 embryonic genotype

In females exposed to 20 mg/kg, ≈38% of p53 positive embryos had digit anomalies of the forelimbs and nearly 100% had digit anomalies of the hindlimbs (Table 3). At the same time, no p53 negative embryos with malformed forelimbs were detected and ≈14% of the embryos had digit anomalies of the hindlimbs. Limb reduction anomalies were also observed only in the hindlimbs of p53 positive embryos (Table 3). The same relationship between teratologic limb phenotypes and p53 embryonic genotype was registered when mice were exposed to other doses of CP. Thus, the limbs of p53 negative embryos of females exposed to 12.5 mg/kg CP were normal, whereas 10% and ≈40% of p53 positive embryos had digit anomalies of the forelimbs and hindlimbs, respectively. When mice were exposed to 40 mg/kg CP, digit anomalies of the forelimbs were observed in all p53 positive embryos and in ≈71% of p53 negative embryos. Limb reduction anomalies of the hindlimbs were also observed in 70% of p53 negative embryos and all p53 positive embryos. Thus, these results demonstrate that the limbs of p53 positive embryos are more sensitive to CP than the limbs of p53 knockout embryos, with differential teratologic sensitivity of the fore- and hindlimbs being independent on p53 embryonic genotype.

**Table 3:** Effects of p53 on CP-induced limb phenotype (dose of 20 mg/kg)

Tested embryonic structure	p53 genotype	Number of tested embryos	Number of embryos exhibiting:	
			Digit anomalies	Limb reduction anomalies
Forelimbs	+/+ and +/-	64	24(37.5%)	0
	-/-	28	0*	0

Hindlimbs	+/+ and +/-	64	62 (96.9%)	24 (37.5%)
	-/-	28	4 (14.3%)*	0*

Differences between p53 positive and p53 negative embryos are statistically significant (the Fisher exact test, p < 0.05).

### Influence of CP on miRNA-34a expression

**Figure 1:**
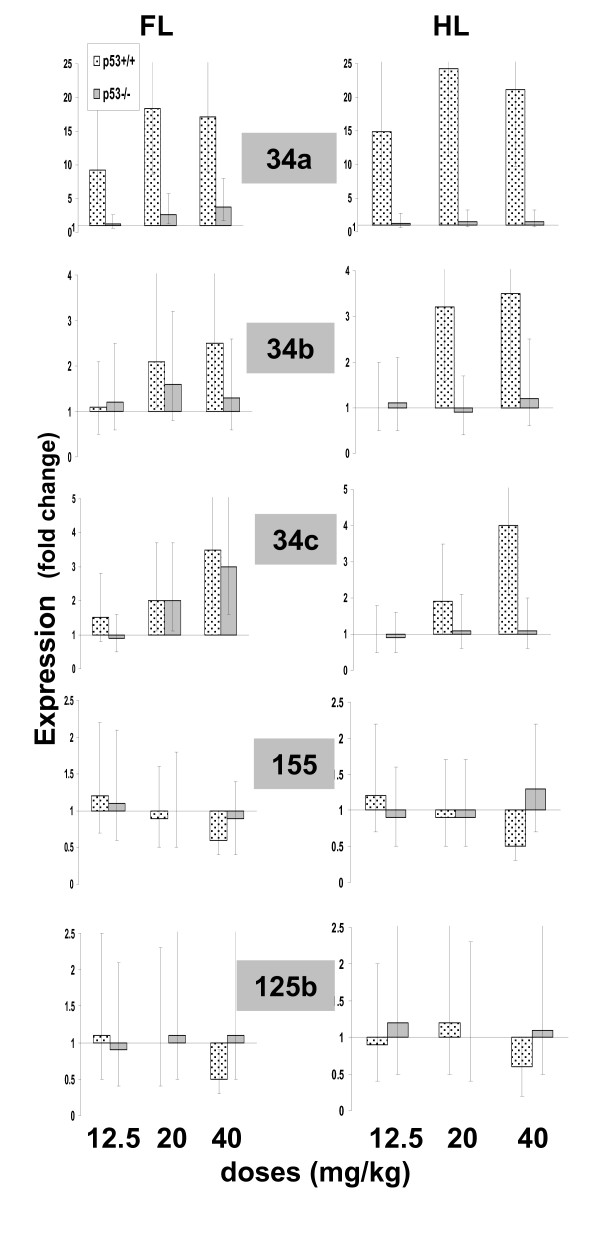
**Expression of miRNA-34, miRNA-125b and miRNA-155 in the forelimbs (FL) and hindlimbs (HL) of p53^+/+ ^and p53^+/+ ^embryos of mice exposed to different doses of CP**. Samples were run in triplicate. Relative levels of the miRNAs were calculated using the 2^-ΔΔCT ^method (U6B was used as an endogenous control). Results were analyzed statistically using the GT2 test for multiple comparisons (n = 4, k = 12) and presented as95% comparison intervals for the means. Means with intervals that do not overlap are significantly different. Means with intervals, which do not reach 1 (the level of expression in controls), differ significantly from controls.

CP at a dose of 12.5 mg/kg induced robust miRNA-34a expression in the fore- and hindlimbs of p53^+/+ ^but not in the limbs of p53^-/- ^embryos (Figure 1). No statistically significant differences in miRNA-34a levels registered in the fore- and hindlimbs were observed. When females were treated with 20 mg/kg CP, p53^+/+ ^embryos exhibited statistically insignificant but almost two-fold increase in the level of miRNA-34a expression as compared to that registered in the embryos exposed to the lower dose of CP. Remarkably, this dose of CP also induced 2-fold increase in miRNA-34a expression in the forelimbs of p53^-/- ^embryos. In the limbs of embryos exposed to 40 mg/kg CP the expression pattern of the miR was identical to that registered in the limbs of embryos exposed to 20 mg/kg CP (Figure 1). No differences in the levels of miRNA-34a expression were observed in fore- and hindlimbs of control p53^+/+ ^and p53^-/- ^embryos (data not presented).

### Influence of CP on miRNA-34b and miRNA-34c expression

The expression of miRNA-34b and miRNA-34c was not altered in the limbs of embryos exposed to 12.5 mg/kg CP (Figure 1). When females were treated with 20 mg/kg CP, the level of miRNA-34b and miRNA-34c expression in the limbs of p53^+/+ ^embryos were statistically significantly higher than that in the limbs of controls but obviously lower than miRNA-34a levels. Besides, this dose of CP resulted in a statistically significant increase in miRNA-34c expression in the forelimbs of p53^-/- ^embryos. Finally, the expression pattern of these miRNAs in the limbs of embryos exposed to 40 mg/kg CP did not differ from that registered in the limbs of embryos exposed to 20 mg/kg CP (Figure 1). No differences in the levels of miRNA-34b and miRNA-34c expression were observed in fore- and hindlimbs of control p53^+/+ ^and p53^-/- ^embryos (data not presented).

### Influence of CP on miRNA-155 and miRNA-125b expression

The only observed effect on miRNA-155 and miRNA-125b expression was in the limbs of p53^+/+ ^embryos exposed to 40 mg/kg CP. A 1.7 - 2-fold decrease in the expression level of these miRNAs was registered (Figure 1). No differences in the levels of miRNA-155 and miRNA-125b expression were observed in fore- and hindlimbs of control p53^+/+ ^and p53^-/- ^embryos (data not presented).

## Discussion

The objectives of this study were formulated as follows: 1) to evaluate whether CP-induced teratogenic insult alters the expression of several miRNAs (miRNA-34, miRNA-125b and miRNA-155) in mouse embryonic limbs and to what extent these alterations are mediated by p53; and 2) to estimate how CP-induced alterations in the expression of the miRNAs correlates with CP-induced limb phenotypes. We observed that the expression of all tested miRNA-34 family members was elevated in the limbs of CP-treated p53^+/+^embryos. These data are in agreement with those obtained in earlier studies that addressed the effect of p53 on miRNA-34 expression in mice exposed to ionizing radiation [29,30]. Interestingly, miRNA-34a was identified as being strongly regulated by p53 regardless of cell type or stress [31], and in our study, the magnitude of CP-induced activation of miRNA-34a in the limbs of p53^+/+ ^embryos was also significantly higher than that of miRNA-34b and miRNA-34c. Yet, in the studies cited above and studies addressing the expression of miRNA-34a only [32], DNA-damaging stress-induced activation of the miRNA-34 family was found to be highly p53 dependent. By contrast, in our work, miRNA-34a and miRNA-34c were found to be activated not only in the limbs of CP-treated p53^+/+ ^embryos but also in the forelimbs of CP-treated p53 knockout embryos. As the expression level of miRNA-34a was significantly higher in the former, this observation does not question the role of p53 as a bona fide mediator of CP -induced activation of miRNA-34a. Interestingly, however, miRNA-34c expression was practically identical in the limbs of CP-treated p53^+/+ ^and p53^-/- ^embryos. Altogether, our data demonstrate for the first time that in some embryonic tissues p53-independent mechanisms may exist, contributing to teratogen-induced activation of miRNA-34a and miRNA-34c. (After the manuscript was submitted for publication, evidence appeared demonstrating p53-independent activation of miRNA-34a in primary human TIG3 fibroblasts during oncogene-induced senescence [33]).

Whereas the levels of miRNA-34 increased in CP-treated embryos, miRNA-125b and miRNA-155 levels clearly tended to decrease in the limbs of p53^+/+^embryos exposed to 40 mg/kg CP. We have not encountered any publications addressing the expression of miRNA-155 in teratogen-treated embryos. As to miRNA-125b, its expression was found to be decreased in embryos of female rats treated with such a teratogen as retinoic acid [34]. Two other teratogens, ionizing radiation and camptothecin [35], have been shown to suppress miRNA-125b expression in zebrafish embryos [36]. Our results concur with the above observations and, in parallel, for the first time, demonstrate that teratogen-induced suppression of miRNA-125b and miRNA-155 may be p53 dependent.

The analysis of studies addressing the biological activities of miRNA-34, miRNA-125b and miRNA-155 strongly suggests that all tested miRNAs may be involved in the mechanism of determining the response of the embryo to CP-induced teratogenic stimuli. Indeed, apoptosis and suppression of cell proliferation are key intermediate cellular events in CP teratogenesis [16,22,28], and our studies indicate that p53 acts to intensify these events [16]. The miRNA-34 is activated by p53 being able to mediate p53-induced proapoptotic and antiproliferative effects. Besides, recent observations suggest the ability of miRNA-34a to indirectly increase p53 activity [37]. Finally, we observed miRNA-34a and miRNA-34c activation in the forelimbs of CP-treated p53 knockout embryos. In this context, it is noteworthy that p53-independent mechanisms of miRNA-34a-induced apoptosis were suggested [38]. It also supposes that miRNA-34a may act in cooperation with miRNA-34c, which is predicted to have the same seed regions and mRNA targets [31]. A recent study reinforces this proposition: cells exposed to a DNA double-strand break agent (doxorubicin: DOX) demonstrated that only simultaneous inhibition or forced expression of miRNA-34a and miRNA-34c resulted in the inhibition or induction of DOX-mediated apoptosis [39]. These observations seemingly suggest the possibility of miRNA-34a and miRNA-34c mediating CP-induced apoptosis in the forelimbs of p53^-/- ^embryos. On the other hand, unlike miRNA-34 ability to act in concert with p53, miRNA-125b and miRNA-155 seem to have the potential to function as inhibitors of CP-induced p53-mediated apoptosis. Indeed, it has been shown that miRNA-155 suppresses the expression of the tumor protein 53-induced nuclear protein 1, which induces apoptosis and cell arrest in several cell lines [40]. Furthermore, there exist data implying that miRNA-155 can be a blocker of caspase 3 activation [24,25], which in turn is activated by teratogenic doses of CP in a p53-dependent fashion [16]. As to miRNA-125b, it was recently suggested to be a bona fide negative regulator of p53 in zebrafish and humans [36]. Finally, as miRNA-155 and miRNA-125b exhibited similar expression patterns, an intriguing question was whether CP-induced suppression of these miRNAs can intensify p53-mediated apoptosis employing shared targets of these miRNAs. To address this question, we employed three independent miR target prediction databases: TatgetScan [41] TargetRank [42] and PITA [43] and then crossed these data to find high-confidence putative targets. As a result, five genes, such as SOX11, KCNA1, E2F2, ETS1 and MAP3K10, were predicted as common targets for miRNA-125b and miRNA-155. Two of them have the potential to intensify p53-mediated apoptosis. The first one is ETS1, which is suggested to be required for p53 transcriptional activity [44]. The second, MAP3K10, was shown to induce P53-mediated apoptosis following phosphorylation [45].

In the light of the above data, the activation of miRNA-34 and suppression of miRNA-125b and miRNA-155 in the limbs of CP-treated embryos may be suggested as pathogenetic events in CP-induced apoptosis and, hence, CP-induced limb dysmorphogenesis. Yet, if we analyze how the changes in the expression profile of the miRNAs correlate with the severity of the CP-induced teratologic phenotype, the above suggestion is compromised. Indeed, in the limbs of embryos exposed to 20 and 40 mg/kg CP, the expression profile of these miRNAs was identical although the higher dose affected limb development much stronger. Also, at all dose levels, the hindlimbs of the embryos were more severely affected than the forelimbs. Yet, the levels of miRNA-34, miRNA-125b and miRNA-155 expression were found to be practically identical in the hindlimbs and the forelimbs of p53-positive embryos. In addition, an elevated expression of miRNA-34a and miRNA-34c was detected only in less teratologically sensitive forelimbs of p53 negative embryos. Together, these findings scarcely allow suggesting that these miRNAs mediate CP-induced limb dysmorphogenesis. If it is the case, what is a possible explanation of this discrepancy?

A plausible explanation may be found if we bear in mind the concept proclaiming that the main function of miRNAs is to confer robustness to developmental programs [46-48]. Within this concept, the main function of miRNAs positively regulating apoptosis in the embryo may be formulated as preventing the birth of malformed offspring. These miRNAs may perform this function by either mediating an "adaptive apoptosis" which will contribute to the renewal of teratogen-targeted cell populations by promoting the removal of injured cells or activating teratogen-induced apoptosis in order to kill severely malformed embryos. If this is the case, a model may be proposed in which the activation of some proapoptotic miRNAs may represent an adaptive response to teratogenic apoptotic stimuli, whereas other proapoptotic miRNAs are activated and/or antiapoptotic miRNAs are suppressed to strengthen teratogen-induced apoptosis. This model does not contradict results obtained from teratological studies implying that proapoptotic signaling may be indispensable for embryo protection again teratogenic stress [11]. It is also in agreement with the suggestion that miRNAs are ideal candidates for the safeguarding of organisms during environmental stresses [48,49]. Finally, within the context of this model, some of the results can be partially explained. Indeed, only miRNA-34a was activated in p53^+/+ ^embryos exposed to 12.5 mg/kg CP. Of note, this miRNA is suggested to be the most stress-sensitive member of the miRNA-34 family [31], whereas the dose of 12.5 mg/kg is a threshold teratogenic dose for these embryos. A further increase in miRNA-34a expression and an elevated expression of miRNA-34b and miRNA-34c were detected in p53^+/+ ^embryos exposed to 20 mg/kg, a dose, to which a part of embryos are still able to resist. At the same time, the expression pattern of the miRNA-34 did not change in embryos exposed to a dose of 40 mg/kg severely affecting all embryos. Instead, the expression of miRNA-125b and miRNA-155 having the potential to negatively regulate the p53-mediated proapoptotic signaling was suppressed. Remarkably, the observation that the expression pattern of all tested miRNAs was identical in the forelimbs and hindlimbs of CP-treated p53^+/+ ^embryos does not contradict the proposed model.

## Conclusion

This study demonstrates that teratogen-induced limb dysmorphogenesis may be associated with alterations in miRNA-34, miRNA-125b and miRNA-155 expression. It also suggests for the first time that in some embryonic tissues p53-independent mechanisms may exist, contributing to teratogen-induced activation of miRNA-34a and miRNA-34c, whereas teratogen-induced suppression of miRNA-125b and miRNA-155 expression may be p53 dependent. Finally, the analysis of correlations between the expression pattern of the tested miRNAs and CP-induced limb phenotypes allows us to hypothesize that miRNAs regulating apoptosis may differ from each other with respect to their functional role in teratogenesis. Some miRNAs may act to protect embryos, whereas other miRNAs may boost a teratogen-induced process of maldevelopment thus inducing embryonic death. This hypothesis should be taken into account in further studies addressing the role of miRNAs in teratogenesis.

## Methods

### Animals and CP treatment

Breeding pairs of p53 knockout mice bearing a mutation deleting 40% of the p53- coding region and completely blocking production of p53 protein [50] were received as a gift from Prof. Moshe Oren (Weizmann Institute of Science, Israel), and now a colony of these mice is being maintained in Tel Aviv University Animal Facility on a 14 h light: 10 h darkness cycle by crossing p53^+/- ^females with p53^-/- ^males. To obtain pregnancies, 3-month-old p53^+/- ^females were caged with p53^+/- ^males for 3 h, from 0700 to 1000 h (darkness), and the presence of a vaginal plug (1100 h) was designated as day 1 of gestation. CP (Sigma) was injected intraperitoneally at 1000 h of day 12 of gestation at 12.5, 20 or 40 mg/kg CP (in 0.5 ml saline/20 g body weight). Pregnant females injected with saline (0.5 ml/20 g bodyweight) were used as a control throughout the study. Animal experiments were approved by the Ethics Committee for Animal Use of Tel Aviv University.

### Genotyping

Genotyping of embryos was performed as described elsewhere [16]. Briefly, DNA was extracted from the amnion and PCR was performed using PCR mix (Promega), DNA, and 3 primers (Sigma): 5'-ACAGCGTGGTGGTACCTTAT-3', 5'-TATACTCAGAGCCGGCCT-3', and 5'-CTATCAGGACATAGCGTTGG-3' [50] under the following conditions: initial preheating at 94 8C for 3 min followed by 30 cycles of the following three steps: 1) denaturing (94 8C) for 30 s, 2) annealing (55 8C) for 30 s, and 3) extension (72 8C) for 1 min followed by 3 min at 72 8C. PCR products were diluted in a DNA-loading buffer and loaded on 1.2% agarose gel diluted in a TBE buffer with ethidium bromide (Sigma).

### Teratological testing

The spectrum of external anomalies induced by CP in mice is very wide [51]. Our choice of the forelimbs and hindlimbs as targets was based on our and other studies demonstrating that these organs are extremely but differentially sensitive to the teratogen (the hindlimbs exhibit higher sensitivity) [22,52,53]. To evaluate the CP-induced limb teratologic phenotypes, females were sacrificed by cervical dislocation on day 16 of gestation, the uteri were removed and implantation sites, resorptions and live embryos were recorded. Live fetuses were fixed in Bouin's solution, and examined visually for structural anomalies such as adactyly, ectrodactyly, syndactyly (digit anomalies) as well as more severe limb reduction anomalies such as, apodia, hemimelia and amelia. As we previously showed, the response of p53^-/- ^embryos to CP-induced teratogenic stimuli strikingly differs from that demonstrated by p53^+/+ ^and p53^+/- ^embryos, which, in turn, are equally sensitive to the teratogen [16]. Therefore, the results characterizing teratogenic response of p53^+/+ ^and p53^+/- ^embryos were pooled.

### miRNA analysis

Evaluation of the expression of miRNA-34, miRNA-125b and miRNA-155 was performed in the fore- and hindlimbs of p53^+/+ ^and p53^-/- ^embryos collected 24 hours after CP injection. The choice of this time point was based on our previous studies [16,23] that revealed that embryos tested 24 hours after CP treatment exhibited not only prominent apoptosis but also the highest levels of the expression of such proapoptotic molecules as caspases 3, 8 and 9, the strongest suppression of DNA-binding activity of an anti-apoptotic molecule, the transcription factor NF-kB, and the lowest levels of the expression of molecules acting in the classical NF-kB signaling pathway such as IKKβ, IkBα, I-kBβ and I-kBε. To obtain a tested sample, embryos collected from five-six litters were pooled. The expression of the miRNAs was tested in four samples obtained for control and experimental groups.

### RNA extraction

Extraction of miRNA-enriched total RNAs was performed using QIAGEN's RNeasy Plus Mini Kit (QUIAGENE, Hilden, Germany) following the manufacturer's protocol with slight modifications. Briefly, a tested embryonic structure was homogenized using RLT lysis buffer mixed with β-mercaptoethanol. The homogenate was transferred to a gDNA Eliminator spin column. Then, the lysate obtained was added with 1.5 volumes of 100% ethanol, transferred to a filter spin column and washed twice in order to increase RNA quality. Finally, RNA was eluted with nuclease-free water and the sample was evaluated for quality and quantity by ND-1000 according to the manufacturer's V3.5 User's Manual for the NanoDrop^® ^ND-1000 Spectrophotometer (NanoDrop Technologies, Wilmington, DE).

### Quantitative Real-Time PCR

In order to test the integrity of the small RNA fraction in our specimens prior to miRNA profiling, we performed quantitative analysis of a non-coding small RNA, U6 RNA as described below. Based on results obtained, it is reasonable to assume that miRNAs are intact and could be accurately quantified in the tested specimens. Real-Time PCR samples were prepared using a TaqMan PCR master mix and specific real-time primers for the tested miRNAs (ABI) according the manufacturer's protocol with slight modifications using ABI 7900HT fast real-time PCR system. Briefly, 7 μl real-time mixture containing 0.5 μl RT product, 0.35 μl real-time primer (including a probe, the forward and reverse primers) and 1× TaqMan Universal PCR Master Mix was prepared. The mixture was incubated in a 384-well plate at 95°C for 10 min, followed by 40 cycles of 95°C for 15 s and 60°C for 1 min. All reactions were run in triplicates. miRNA expression levels were calculated by using the ABI 7500 Real-Time PCR SDS 1.2 software (ABI). The 2^-ΔΔCT ^method [54] was used to calculate relative expression of the tested miRNAs.

### Statistical analysis

Statistical analysis of the teratological data was performed on a litter basis using the GT2-method for multiple comparisons [55]. For this, values characterizing the proportions of resorptions and malformed fetuses in each litter of each group were transformed to arsine values by Freeman-Tukey's method as described elsewhere [56] and the means and standard errors of these indices were calculated for each group. To look for an association across treatment groups, embryonic genotypes and teratologic phenotypes, the χ^2 ^test was run as described [57]. GT2-method for multiple comparisons was used to analyze statistically data characterizing the expression of the miRNAs. The two-tailed level of significance of differences was equal to 0.05 for all tested parameters.

## Authors' contributions

KG - performed miRNA analysis, participated in p53 genotyping and teratological testing; NM - guided and performed miRNA analysis; NG - performed p53 genotyping and participated in miRNA analysis and teratological testing; EM - miRNA targets prediction and functional analysis; SS and AF- performed teratological testing and interpreted thereof, helped drafting the manuscript; NS - interpreted results of miRNA analysis and drafted the manuscript; VT and AT - conceived the study, planned experiments and wrote the majority of the paper. All authors read and approved the final manuscript.
